# Lumbar puncture for non-HIV-infected non-transplant patients with cryptococcosis: Should it be mandatory for all?

**DOI:** 10.1371/journal.pone.0221657

**Published:** 2019-08-22

**Authors:** Sung-Hsi Huang, Yu-Chung Chuang, Yi-Chien Lee, Chien-Ching Hung, Wang-Huei Sheng, Jen Jen Su, Hsin-Yun Sun, Yee-Chun Chen, Shan-Chwen Chang

**Affiliations:** 1 Department of Internal Medicine, National Taiwan University Hospital Hsin-Chu Branch, Hsin-Chu, Taiwan; 2 Department of Tropical Medicine and Parasitology, National Taiwan University College of Medicine, Taipei, Taiwan; 3 Department of Internal Medicine, National Taiwan University Hospital, Taipei, Taiwan; 4 Department of Internal Medicine, Fu Jen Catholic University Hospital, New Taipei, Taiwan; 5 Department of Neurology, National Taiwan University Hospital, Taipei, Taiwan; 6 School of Medicine, National Taiwan University College of Medicine, Taipei, Taiwan; 7 Graduate Institute of Clinical Pharmacy, National Taiwan University College of Medicine, Taipei, Taiwan; Rutgers University, UNITED STATES

## Abstract

**Background:**

The indications for lumbar puncture in non-HIV-infected, non-transplant (NHNT) patients with cryptococcosis without meningeal signs need to be more fully defined.

**Objectives:**

This study was designed to determine the optimal predictors of central nervous system (CNS) involvement in adult NHNT patients with cryptococcosis.

**Methods:**

The study population consisted of adult NHNT patients with culture-confirmed cryptococcosis who sought care at a university hospital in Taiwan from 2002 to 2016. We used a case-control method to identify the clinical characteristics and laboratory findings associated with CNS involvement in patients who underwent a diagnostic lumbar puncture. In the sensitivity analysis, we included additional control patients who did not undergo lumbar puncture, but were followed beyond 12 months without the development of CNS involvement in the absence of exposure to any fungicidal agents.

**Results:**

We entered 270 NHNT adult patients into the study during the 15-year period. CNS involvement was confirmed in 66 (71.0%) of 93 patients who underwent lumbar puncture. A multivariable analysis revealed that presence of neurological manifestations and elevated serum CRAG titers were independently associated with a 23.97-fold (95% confidence interval [CI], 4.37–182.23) and 1.53-fold (per 2-fold increment, 95% CI 1.26–1.92) increased odds ratio for CNS involvement, respectively. Headache and focal neurologic signs were independently associated with CNS involvement. A cut-off serum CRAG titer of ≥1:64 provided the highest diagnostic performance by Youden index (sensitivity 83% and specificity 65%). Similar findings were noted in the sensitivity analysis including 198 (73%) patients.

**Conclusion:**

Lumbar puncture is indicated for NHNT patients with cryptococcosis who have neurologic manifestations or a serum CRAG titer of ≥1:64.

## Introduction

Central nervous system (CNS) involvement is the most serious complication of cryptococcosis because of its high mortality and debilitating neurologic sequelae among the survivors [1,2]. Treatment of CNS cryptococcosis requires a combination of fungicidal agents during the induction phase and aggressive control of intracranial pressure to improve early survival [2–4].

Many of the recent advances in the management of patients with cryptococcosis have been generated from studies of patients with HIV infection and organ transplant recipients [5]. Non-HIV-infected, non-transplant (NHNT) patients account for a substantial proportion of patients with cryptococcosis [6–9]. Their optimal management is less well defined.

NHNT patients with cryptococcosis are reported to have a lower rate of CNS involvement than HIV-infected patients or organ transplant recipients [6,9]. Nevertheless, they had a higher mortality rate [6–9] and severe neurologic morbidities [10,11] when CNS involvement became evident. The time from presenting symptoms to confirmed diagnosis is also longer in NHNT patients than HIV-infected patients or organ transplant recipients. Several reports have linked this to poorer outcomes [6,9,12].

Cerebrospinal fluid (CSF) analysis is required to confirm or exclude CNS involvement in patients with cryptococcosis. Imaging studies are neither sensitive nor specific [2,3,5]. Routine lumbar puncture is recommended for patients with HIV infection, other severe immunocompromising conditions, and disseminated cryptococcosis with clinical presentations compatible with CNS infection [3,5]. However, there is limited evidence to support this recommendation for NHNT patients with cryptococcosis and compliance to guidelines is reported to be suboptimal [13]. This study was designed to determine the clinical and laboratory predictors of CNS involvement in NHNT patients in order to identify those who would more likely benefit from a lumbar puncture.

## Patients and methods

### Study design and patients

This case-control study was conducted at the National Taiwan University Hospital, a 2,300-bed teaching hospital located in northern Taiwan that provides both primary and tertiary healthcare. We reviewed the records of patients with one or more positive cultures for *Cryptococcus neoformans/Cryptococcus gattii* species complex isolated from any clinical specimens from 2002 to 2016. A standard case recording form was used to collect data on demographic characteristics, predisposing factors, clinical presentations, and laboratory investigations.

Routine care for patients with cryptococcal disease in this hospital during the past 2 decades includes provider-initiated testing and counseling for HIV, determination of serum cryptococcal antigen, and lumbar punctures in accordance with current guidelines [3,5]. The details for management in patients with cryptococcal meningitis [14] and cryptococcemia [15], and control of intracranial pressure [16] have been described in the third version of the Taiwan national guidelines published in 2016 [17]. The study was approved by the Research Ethical Committee of the National Taiwan University Hospital with a waiver of informed consent (register number 201802002RIND). The study was carried out in accordance with the approved ethical guidelines and regulations.

### Definitions

Culture-confirmed cryptococcal disease was defined as either a positive culture for *C*. *neoformans/C*. *gattii* species complex obtained from a sterile site or a positive culture from a non-sterile site plus a compatible clinical presentation. We excluded patients with an incidental positive culture of *C*. *neoformans* from a non-sterile site, who did not have cryptococcosis-relevant signs and symptoms, and had not received antifungal treatment at this hospital.

The primary study population consisted of hospitalized adult NHNT patients with both culture-confirmed cryptococcal disease and CSF data. They were divided into two groups according to the findings of their CSF analyses. Cases consisted of patients with cryptococcosis and CNS involvement. CNS involvement was defined as the presence of either a positive CSF culture for *C*. *neoformans* or a positive CSF CRAG titer ≥1:8. Controls consisted of patients with cryptococcosis without CNS involvement.

For the sensitivity analysis we added patients with cryptococcosis, who did not have a lumbar puncture, to the primary study group because CNS involvement was deemed very unlikely. These patients were treated with azoles or observed without antifungal therapy. None were treated with amphotericin B or flucytosine. They were followed for more than 12 months without development of CNS disease or recurrence of cryptococcosis.

Chronic liver disease was defined as either the presence of serologic markers for chronic hepatitis B or C, or clinical and radiologic findings suggestive of cirrhosis of the liver. Chronic kidney disease was defined as persistence of markers of renal damage such as proteinuria or an estimated glomerular filtration rate (eGFR) below 60 ml/min/1.73m^2^ for at least 3 months. Prolonged steroid exposure was defined as a mean minimum dose of 0.3 mg/kg/day of prednisolone for >3 weeks according to EORTC/MSG consensus [18]. Selected medical conditions recorded in this study included diabetes mellitus, chronic liver and kidney diseases, autoimmune diseases, hematologic or solid-organ malignancies, use of steroids or other immunosuppressant agents, and hypogammaglobulinemia. Neurologic manifestations included headaches, altered mental status, seizures, meningeal signs and focal neurologic signs. Neutropenia and lymphocytopenia were defined as an absolute neutrophil and lymphocyte count of less than 1,500 and 1,000 cells/μL, respectively. Hyponatremia was defined as a serum sodium level of less than 136 mmol/L.

### Laboratory investigations

Cryptococci were identified by standard mycological techniques for *C*. *neoformans/C*. *gattii* species complex as previously described [19]. A previous island-wide surveillance study has shown that *C*. *gattii* accounted for only 4.1% of the clinical cryptococcal isolates [7]. We did not differentiate *C*. *neoformans* from *C*. *gattii* in the present study. Serum cryptococcal antigen (CRAG) assays were performed using the Latex-Cryptococcus Antigen Test (IMMY, Oklahoma, USA) according to the manufacturer’s instructions.

### Statistical analyses

Statistical analyses were performed using R statistics software (Version 3.3.3, the R Foundation). Continuous variables were compared by Student’s t-test or Mann-Whitney U tests. Categorical variables were compared by Fisher’s exact test between the groups with and without CNS involvement. Titers of serum CRAG were log_2_-transformed before analyses. Variables with a *p*-value <0.2 in univariable analyses were entered into a binary logistic regression model for multivariable analysis, where missing values (including 1 serum sodium level and 8 serum CRAG titers) were treated by imputation with means. All tests were two-tailed. A *p*-value of <0.05 was considered significant. The same process of analyses was repeated in the sensitivity analysis.

*Post hoc* analyses of the primary study population were performed to elucidate the correlation between the identified variables and CNS involvement. This included a multivariable analysis of various neurologic manifestations to identify the most relevant neurologic signs and symptoms, a receiver operating characteristic (ROC) curve to demonstrate the diagnostic performance of serum CRAG titers to predict CNS involvement, and calculation of Youden indices [20] to determine the optimal serum CRAG cut-off values.

## Results

**Fig 1:**
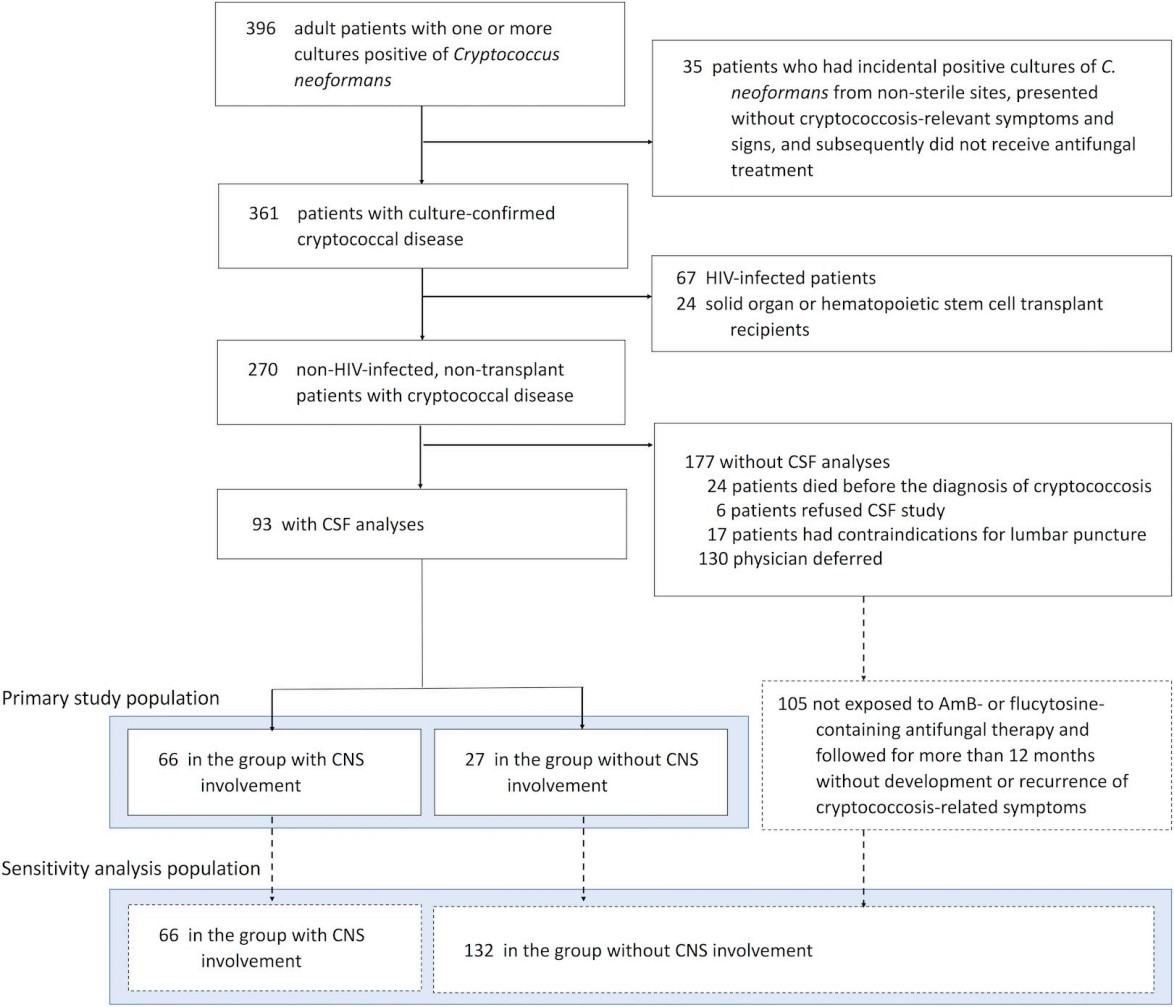
Study flow.

During the 15-year study period, we identified 361 adult patients with culture-confirmed cryptococcal diseases (Fig 1). Most, 270 (74.8%), were NHNT. After appropriate exclusion, shown in Fig 1, 93 of the NHNT patients that had a lumbar puncture were entered into the primary study population. A total of 198 NTHT patients were entered into the sensitivity analysis (66 with CNS involvement and 132 without CNS involvement).

### Characteristics of the primary study population

Ninety-three patients were enrolled in the primary study population. Almost two-thirds were male. The median age was 61 years (interquartile range (IQR) 51–70). Most of the patients (80.6%) had at least one underlying immunocompromising medical condition. The most common conditions were exposure to steroids or other immunosuppressants, diabetes mellitus, malignancies, chronic liver or kidney disease, and autoimmune diseases (Table 1). The clinical characteristics and mortality among the NHNT patients according to whether or not they had a lumbar puncture are shown in S1 Table.

**Table 1:** Baseline characteristics of 93 non-HIV-infected, non-transplant patients with cryptococcosis according to CNS involvement.

	All (N = 93)	With CNS involvement (N = 66)	Without CNS involvement (N = 27)	Univariate analysis		Multivariate analysis ^h^	
				Odds ratio, 95% CI	*p*-value	Odds ratio, 95%CI	*p*-value
Age, median (IQR), years	64 (49, 72)	65 (53, 75)	62 (47, 69)	1.02 (0.99, 1.05)	0.216	—	
Male, n (%)	59 (63.4)	39 (59.1)	20 (74.1)	0.51 (0.16, 1.48)	0.237	—	
Underlying medical condition(s) ^a^, n (%)							
Steroid exposure, any	36 (38.7)	26 (39.4)	10 (37.0)	1.10 (0.40, 3.15)	>0.999	—	
Steroid exposure, prolonged^b^	23 (24.7)	16 (24.2)	7 (25.9)	0.92 (0.30, 3.04)	>0.999	—	
Diabetes mellitus	28 (30.1)	17 (25.8)	11 (40.7)	0.51 (0.18, 1.47)	0.213	—	
Use of immunosuppressants other than steroids^c^	24 (25.8)	19 (28.8)	5 (18.5)	1.77 (0.54, 6.86)	0.435	—	
Autoimmune disease	23 (24.7)	17 (25.8)	6 (22.2)	1.21 (0.38, 4.29)	0.797	—	
Chronic liver disease^d^	22 (23.7)	18 (27.3)	4 (14.8)	2.14 (0.61, 9.69)	0.284	—	
Chronic kidney disease^e^	19 (20.4)	14 (21.2)	5 (18.5)	1.18 (0.35, 4.72)	>0.999	—	
Solid organ malignancy	18 (19.4)	11 (16.7)	7 (25.9)	0.58 (0.17, 2.01)	0.387	—	
Hematologic malignancy	10 (10.8)	8 (12.1)	2 (7.4)	1.72 (0.31, 17.71)	0.718	—	
Absence of immunocompromising conditions^f^	18 (19.4)	12 (18.2)	6 (22.2)	0.78 (0.23, 2.87)	0.773	—	
Symptoms and signs, n (%)							
Fever	61 (65.6)	45 (68.2)	16 (59.3)	1.47 (0.52, 4.02)	0.474	—	
Any neurologic manifestations^g^	79 (84.9)	63 (95.5)	16 (59.3)	13.89 (3.19, 86.62)	<0.001	23.97 (4.37, 182.23)	<0.001
Laboratory investigations							
WBC, k/μL, median (IQR)	7.80 (5.93, 9.98)	7.76 (5.22, 10.42)	7.80 (6.45, 9.68)	1.00 (0.94, 1.08)	0.732	—	
Neutropenia, n (%)	4 (4.3)	2 (3.0)	2 (7.4)	0.40 (0.03, 5.73)	0.577	—	
Lymphocytopenia, n (%)	47 (50.5)	37 (56.1)	10 (37.0)	2.15 (0.79, 6.12)	0.114	0.63 (0.16, 1.92)	0.483
Hyponatremia, n (%) (N = 92)	48 (52.1)	38 (57.6)	10 (38.5)	2.15 (0.78, 6.18)	0.111	1.21 (0.35, 4.13)	0.759
Cryptococcemia, n (%)	33 (35.5)	25 (37.9)	8 (29.6)	1.44 (0.51, 4.40)	0.485	—	
Positive culture(s) from extrapulmonary, extracranial sites, n (%)	17 (18.3)	9 (13.6)	8 (29.6)	0.38 (0.11, 1.30)	0.083	0.41 (0.09, 1.78)	0.223
SCRAG titer, median (IQR) (N = 85)	512 (32, ≥1024)	≥1024 (64, ≥1024)	32 (4, 128)	1.41 (1.20, 1.69)^i^	<0.001	1.53 (1.26, 1.92)^i^	<0.001
SCRAG ≥ 1:64, n (%)	58 (68.2)	49 (83.1)	9 (34.6)	8.94 (2.87, 30.51)	<0.001	—	

^a^ Medical conditions are presented for 5 or more patients with this condition.

^b^ Prolonged steroid exposure was defined as a minimum dose of 0.3 mg/kg/day of prednisolone for more than 3 weeks according to EORTC/MSG consensus, 2008.

^c^ Recorded immunosuppressants other than steroid included azathioprine, bleomycin, chlorambucil, cisplatin, cyclophosphamide, doxorubicin, fluorouracil, ifosphamide, oxaliplatin, mercaptopurine, methotrexate, mycophenolic acid (MMF), vincristine.

^d^ Chronic liver disease was defined based on evidence of chronic viral hepatitis or the presence of cirrhosis.

^e^ Chronic kidney disease was defined when the estimated glomerular filtration rate (eGFR) below 60 ml/min/1.73m^2^ for at least 3 months.

^f^ Immunocompromising conditions indicated here included diabetes, chronic kidney diseases, cirrhosis of liver, autoimmune diseases, malignant diseases, use of steroid or other immunosuppressants, and hypogammaglobulinemia.

^g^ Neurologic manifestations included headaches, altered mental status, seizures, meningeal signs and focal neurologic signs.

^h^ Variables with *p*-value <0.2 in univariate analysis were included in the multivariate analysis.

^i^ Odds ratio per 2-fold increment of sCRAG.

Abbreviations: CI, confidence interval; CNS, central nervous system; sCARG, serum cryptococcal antigen; WBC, white blood-cell count.

### Case-control analysis

In univariable analysis, patients with CNS involvement were more likely to present with neurological manifestations (95.5% vs 59.3%, *p*<0.001), a higher median serum CRAG titer (≥1:1024 vs 1:32, *p*<0.001) and a higher proportion of serum CRAG titers of ≥1:64 (83.1% vs 34.6%, *p*<0.001). There was no significant difference between the two groups in the frequency of cryptococcemia (37.9% vs 29.6%, *p* = 0.485) or a positive culture from extrapulmonary, extracranial sites (13.6% vs 29.6%, *p* = 0.083).

Variables including neurological manifestations, serum CRAG titers, lymphocytopenia, hyponatremia, and involvement of extrapulmonary, extracranial sites were entered into the multivariable model. The presence of neurologic manifestations and higher serum CRAG titers were found to be independently associated with CNS involvement with an odds ratio of 23.97 (95% confidence interval [CI], 4.37–182.23) and 1.53 per 1-log_2_ serum CRAG titer increment (95% CI 1.26–1.92), respectively (Table 1). The findings were similar in the sensitivity analysis (S2 Table).

### *Post-hoc* analysis

We further explored the associations between CNS involvement and various neurological manifestations. Patients with CNS involvement were more likely to present with headache (54.5%), meningeal signs (21.2%) and focal neurologic signs (39.4%). In multivariable analysis, headaches and focal neurologic signs were independently associated with CNS involvement (odds ratio 12.30 [95% CI 3.09–83.55] and 4.02 [95% CI 1.23–16.01], respectively) (S3 Table). Of note, 3 of 14 patients (21.4%), who had no neurologic manifestations, had CNS involvement.

**Fig 2:**
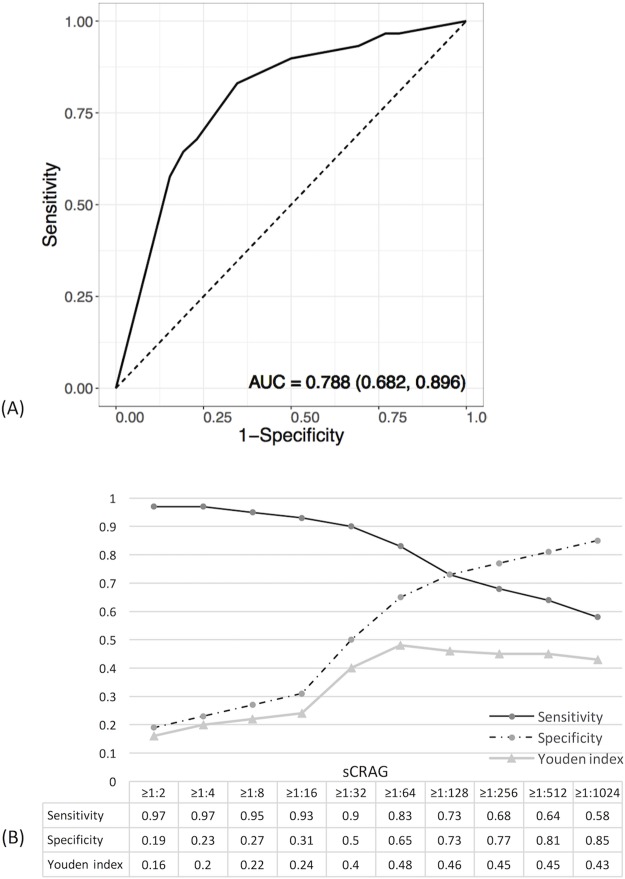
Diagnostic performance of serum cryptococcal antigen (sCRAG) titers to predict CNS involvement in non-HIV-infected, non-transplant patients with cryptococcosis. Panel (A) shows the receiver operating characteristic (ROC) curve, area-under-the-curve (AUC) value of 0.788 (95% confidence interval 0.682 and 0.896). Panel (B) shows the sensitivities, specificities and Youden indices of sCRAG at different cut-off values.

The diagnostic performance of serum CRAG titers for CNS involvement, analyzed by the ROC curve method, revealed that serum CRAG titers had a good discriminative value in predicting CNS involvement. The area-under-the-curve (AUC) was 0.788 (95% C.I. 0.682–0.896) (Fig 2A). A serum CRAG titer of ≥1:64 provided the highest Youden index with a sensitivity of 83% and a specificity of 65% (Fig 2B). Further analysis showed that patients with serum CRAG titers of ≥1:64, compared with those with serum CRAG titers of <1:64, were more likely to have the presence of cryptococcemia (44.8% vs 14.8%, *p* = 0.008), lymphocytopenia (58.6% vs 33.3%, *p* = 0.037) and autoimmune diseases (34.5% vs 11.1%, p = 0.035), but not other immunocompromising conditions.

**Fig 3:**
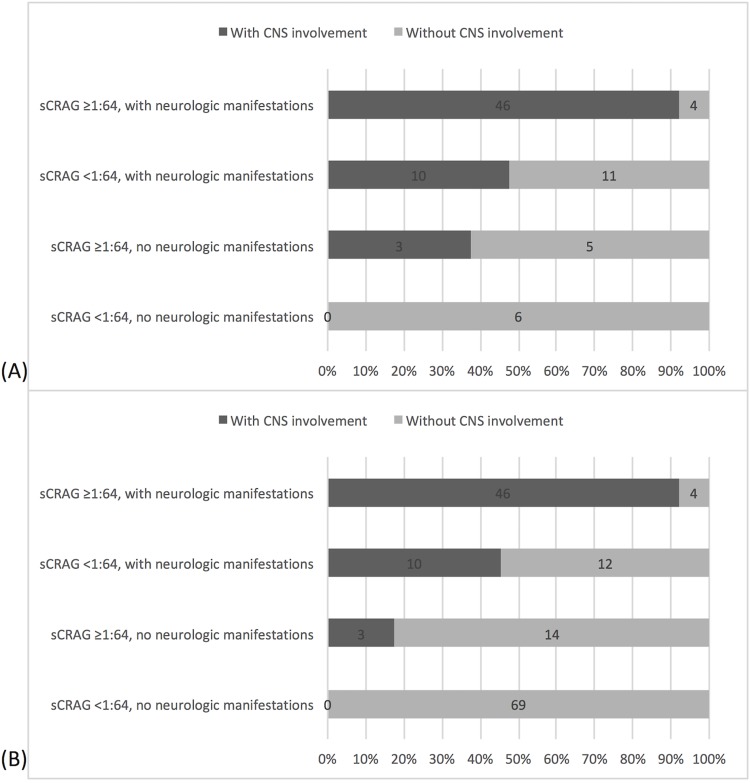
Proportion of patients with central nervous system involvement according to the presence or absence of neurologic manifestations and serum cryptococcal antigen (CRAG) titers of <1:64 and ≥1:64. Panel (A) included 85 patients from the primary study population and had both serum CRAG tests and neurological assessment. Panel (B) included 158 patients from the sensitivity analysis population who had both serum CRAG tests and neurological assessment.

The effect of combinations of serum CRAG ≥1:64 or <1:64 with or without neurological manifestations on the proportion of CNS involvement is shown in Fig 3. The highest proportion (92%, 46/50) was noted with the combination of serum CRAG ≥1:64 and neurological manifestations. Patients with neither of these findings did not have CNS involvement in either the primary or sensitivity study populations.

## Discussion

This large, retrospective study, conducted a teaching hospital in Taiwan, was designed to determine the optimal indicators for lumbar puncture in non-HIV non-transplant patients (NHNT) with cryptococcosis. NHNT accounted for almost three quarters of patients with confirmed cryptococcal diseases at the hospital. Most patients (80.6%) had an underlying immunocompromising medical condition. We found that the presence of neurologic manifestations and elevated serum CRAG titers was independently associated with CNS involvement. A serum CRAG titer ≥1:64 provided the highest diagnostic value. Lower titers, despite their higher sensitivity and low specificity, should not be dismissed in the presence of neurologic manifestations. Characteristic signs of meningeal inflammation were observed in only 21.2% of our patients with CNS cryptococcosis. Similar findings have been reported previously [15,21]. Thus, a substantial number of patients would have been missed if lumbar punctures were deferred based solely on the lack of meningeal signs [14].

A strong correlation between the CSF CRAG titers and CSF cryptococcal colony-forming units has been reported [22]. Serum CRAG titers have also been shown to be associated with extrapulmonary involvement in patients with pulmonary cryptococcosis [21,23]. Serum CRAG titers may reflect the magnitude of the fungal burden. Serum CRAG titers have also been shown to be useful to predict CNS involvement in HIV-infected patients and transplant recipients [23,24]. The serum CRAG assay may be unreliable occasionally. False-positive results may be caused by the prozone phenomenon. Infection with rare species of *Cryptococcus* or those with thin capsules may also result in falsely low or negative CRAG assays despite positive blood or CSF cultures [25,26].

**Fig 4:**
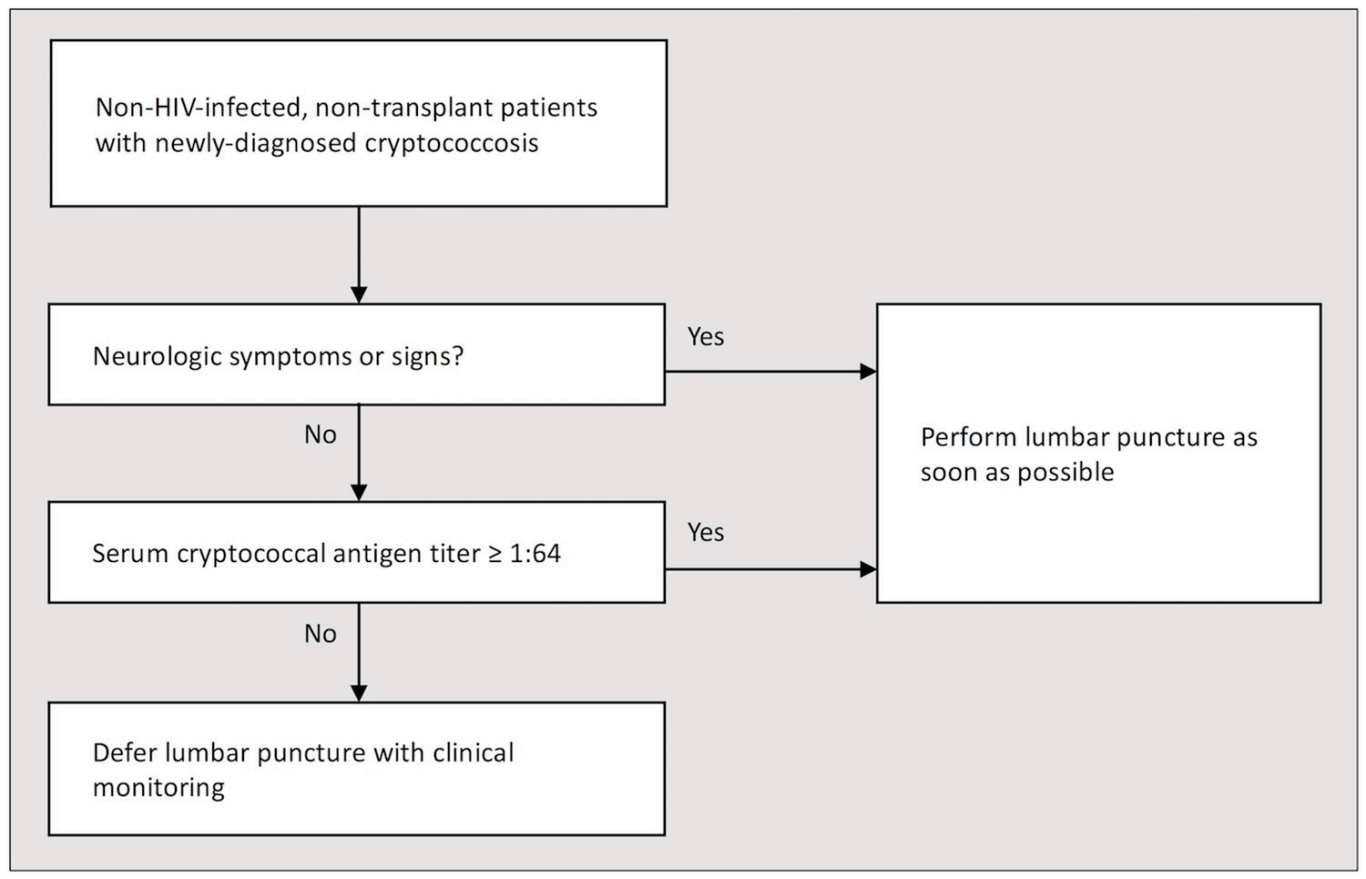
Proposed clinical algorithm to decide whether or not to perform a lumbar puncture in non-HIV-infected, non-transplant patients with newly diagnosed cryptococcosis.

Based on the findings of the current and similar studies in patients with HIV infection and transplantation, we developed an algorithm to facilitate decision-making to perform a lumbar puncture in NHNT patients with newly diagnosed cryptococcosis (Fig 4). The presence of any neurologic manifestation or serum CRAG titers of ≥1:64 should prompt performance of a lumbar puncture to detect potential CNS involvement. The procedure may be deferred with careful observations in patients without neurologic manifestations and serum CRAG titers of <1:64.

The strengths of this study are the large number of patients entered over a 15-year period at a large, referral teaching hospital with an electronic medical records system. The sensitivity analysis included a large number of patients who did not undergo a lumbar puncture. This provided further support for the findings of the case-control study of patients that had a lumbar puncture. The weaknesses of this study are similar to those inherent in all retrospective studies that may lose or misdiagnose some patients for a variety of reasons. NHNT patients with cryptococcosis are a heterogeneous group with a wide variety of underlying immunocompromising conditions. Recent studies have shown that novel acquired immunodeficiencies, such as idiopathic CD4 lymphocytopenia [27] and anti-granulocyte macrophage colony-stimulating factor (anti-GM-CSF) [28], are strongly associated with cryptococcosis. We did not evaluate the influence of these diseases.

In conclusion, we found that neurological manifestations or serum CRAG titers of ≥1:64, alone or in combination, are strong predictors of CNS involvement in NHNT patients with cryptococcosis and are indications for a lumbar puncture. In the absence of these findings lumbar puncture could be deferred provided that the patients continue to be monitored for meningeal signs and serum CRAG as indicated by their course.

## Supporting information

S1 Table Baseline characteristics of 270 non-HIV-infected, non-transplant patients with cryptococcosis. (DOCX)

S2 Table Baseline characteristics of 198 non-HIV-infected, non-transplant patients with cryptococcosis by CNS involvement and the comparison between those with and without CNS involvement by univariable and multivariable analyses. (DOCX)

S3 Table Association of various neurologic manifestations and CNS involvement among non-HIV-infected, non-transplant patients with cryptococcosis by univariable and multivariable analyses. (DOCX)
