# Supplementary material for: Lumbar puncture for non-HIV-infected non-transplant patients with cryptococcosis: Should it be mandatory for all?
Source: PLoS One. 2019 Aug 22;14(8):e0221657. doi: 10.1371/journal.pone.0221657 (PMC6705819; doi:10.1371/journal.pone.0221657)
Supplement: S1 Table — (DOCX) [file pone.0221657.s001.docx]

S1 Table. Baseline characteristics of 270 non-HIV-infected, non-transplant patients with cryptococcosis.

Patients who underwent lumbar puncture were compared to those who did not using Fisher’s exact test or Mann-Whitney U test for statistical analysis. The 133 patients whose physicians deferred a lumbar puncture had mild symptoms, low rates of systemic or neurologic manifestations, lower rates of cryptococcemia and mortality, and lower serum cryptococcal antigens than those who underwent lumbar puncture. Patients who did not undergo a lumber puncture because of early mortality or contraindications were more likely to have chronic liver diseases and cryptococcemia. The mortality rates were high among those who refused or were contraindicated for lumbar puncture.

|  | Patients underwent lumbar puncture  (N=93) | Patients did not undergo lumbar puncture | | | |
| --- | --- | --- | --- | --- | --- |
|  |  | Physician defer  (N=133) | Early mortality^h^  (N=24) | Contraindicated  (N=13) | Patient refusal  (N=7) |
| Age, median (IQR), years, | 61 (51, 70) | 59 (51, 68) | 62 (56, 75) | 57 (61, 67) | 69 (52, 73) |
| Male, n (%) | 59 (63.4) | 80 (60.2) | 12 (50.0) | 8 (61.5) | 3 (42.9) |
| Underlying medical condition(s) ^a^, n (%) |  |  |  |  |  |
| Steroid exposure, any | 36 (38.7) | 21 (15.8)*** | 15 (62.5)* | 5 (38.5) | 1 (14.3) |
| Steroid exposure, prolonged^b^ | 23 (24.7) | 2 (1.5)*** | 6 (25.0) | 2 (15.4) | 0 |
| Diabetes mellitus | 28 (30.1) | 31 (23.3) | 6 (25.0) | 2 (15.4) | 3 (42.9) |
| Immunosuppressants other than steroids^c^ | 24 (25.8) | 13 (9.8)** | 7 (29.2) | 3 (23.1) | 2 (28.6) |
| Autoimmune disease | 23 (24.7) | 15 (11.3)* | 9 (37.5) | 3 (23.1) | 0 |
| Chronic liver disease^d^ | 22 (23.7) | 26 (19.5) | 14 (58.3)** | 8 (61.5)** | 5 (71.4)* |
| Chronic kidney disease^e^ | 19 (20.4) | 17 (12.8) | 10 (41.7) | 3 (23.1) | 2 (28.6) |
| Solid organ malignancy | 60 (30.2) | 52 (39.1)*** | 8 (33.3) | 0.28 (0.03, 2.09) | 3 (42.9) |
| Hematologic malignancy | 10 (10.8) | 6 (6.8) | 0 | 0 | 0 |
| Absence of immunocompromising conditions^f^ | 18 (19.4) | 35 (26.3) | 1 (4.2) | 0 | 0 |
| Symptoms and signs, n (%) |  |  |  |  |  |
| Fever | 61 (65.6) | 17 (12.8)*** | 16 (66.7) | 9 (69.2) | 5 (71.4) |
| Any neurologic manifestations^g^ | 79 (84.9) | 8 (6.0)*** | 15 (62.5)* | 7 (53.8)* | 2 (28.6)* |
| Laboratory investigations |  |  |  |  |  |
| WBC, median (IQR) x 10^3^/μL, | 6.64 (5.07, 8.77) | 6.07 (5.04, 7.68)*** | 15.30 (10.29, 22.26)*** | 8.03 (3.66, 8.77) | 13.06 (7.79, 13.94) |
| Neutropenia, n (%) | 4 (4.3) | 7/130 (5.4) | 1 (4.2) | 1 (7.7) | 0 |
| Lymphocytopenia, n (%) | 47 (50.5) | 18/130 (13.8)*** | 14 (58.3) | 11 (84.6)* | 5 (71.4) |
| Hyponatremia, n (%) | 48 (52.2) | 18/124 (14.5)*** | 10 (41.7) | 7 (53.8) | 4 (57.1) |
| Cryptococcemia, n (%) | 33 (35.5) | 4 (10.8)** | 22 (91.7)*** | 11 (84.6)** | 2 (28.6) |
| Positive culture(s) from extrapulmonary,  extracranial sites, n (%) | 17 (18.3) | 13 (9.8) | 4 (16.7) | 4 (30.8) | 3 (42.9) |
| SCRAG titer, median (IQR) | 16 (0, ≥1024) | <2 (<2, 4)*** | ≥1024 (0, ≥1024) | 128 (32, ≥1024) | 256 (32, ≥1024) |
| Outcome |  |  |  |  |  |
| 14-day mortality, n/N (%) | 17/91 (18.7) | 1/132 (0.7)*** | 24/24 (100)*** | 6/13 (46.2)* | 1/6 (16.7) |
| 3-month mortality, n/N (%) | 35/88 (39.8) | 8/126 (6.3)*** | 24/24 (100)*** | 10/13 (76.9)* | 4/5 (80.0) |
| 12-month mortality, n/N (%) | 40/81 (49.4) | 10 /116 (8.6)*** | 24/24 (100)*** | 11/13 (84.6)* | 4/5 (80.0) |
| Follow-up duration, day, median (IQR) | 222 (20, 1368) | 1127 (463, 2093)*** | 3 (1, 5)*** | 15 (11, 30)** | 28 (12, 37) |

^a^ Medical conditions presented by 5 or more patients were provided.

^b^ Prolonged steroid exposure was defined as a minimum dose of 0.3 mg/kg/day of prednisolone for more than 3 weeks according to EORTC/MSG consensus, 2008.

^c^ Immunosuppressants other than steroid included azathioprine, bleomycin, chlorambucil, cisplatin, cyclophosphamide, doxorubicin, fluorouracil, ifosphamide, oxaliplatin, mercaptopurine, methotrexate, mycophenolic acid (MMF), vincristine.

^d^ Chronic liver disease was defined when there was evidence of chronic viral hepatitis or the presence of cirrhosis.

^e^ Chronic kidney disease was defined when there was evidence of kidney damage or estimated glomerular filtration rate (eGFR) below 60 ml/min/1.73m^2^ for at least 3 months according to KDIGO guideline, 2012.

^f^ Immunocompromising conditions indicated here included diabetes, chronic kidney diseases, cirrhosis of liver, autoimmune diseases, malignant diseases, use of steroid or other immunosuppressants, and hypogammaglobulinemia.

^g^ Neurologic manifestations included headaches, altered mental status, seizures, meningeal signs and focal neurologic signs.

^h^ Early mortality indicated that patients died before the confirmational positive cultures of *Cryptococcus neoformans*.

* *p*-value <0.05

** *p*-value <0.01

*** *p*-value <0.001

Abbreviations: CI, confidence interval; CNS, central nervous system; sCARG, serum cryptococcal antigen titer; WBC, white blood cell count.
