# Supplementary material for: Lumbar puncture for non-HIV-infected non-transplant patients with cryptococcosis: Should it be mandatory for all?
Source: PLoS One. 2019 Aug 22;14(8):e0221657. doi: 10.1371/journal.pone.0221657 (PMC6705819; doi:10.1371/journal.pone.0221657)
Supplement: S2 Table — (DOCX) [file pone.0221657.s002.docx]

S2 Table. Baseline characteristics of 198 non-HIV-infected, non-transplant patients with cryptococcosis by CNS involvement and the comparison between those with and without CNS involvement by univariable and multivariable analyses.

Of the 132 patients in the without CNS involvement group, 105 did not have receive lumbar puncture. They were not exposed to any amphotericin B-containing or flucytosine-containing antifungal therapy after diagnoses of cryptococcosis and were observed for more than 12 months without evidence of development or recurrence of any cryptococcosis-related symptoms or signs.

|  | All  (N=198) | With CNS involvement  (N=66) | Without CNS involvement^h^  (N=132) | Univariate analysis | | Multivariate analysis ^i^ | |
| --- | --- | --- | --- | --- | --- | --- | --- |
|  |  |  |  | Odds ratio, 95% CI | *p*-value | Odds ratio, 95%CI | *p*-value |
| Age, years, median (IQR) | 61 (51, 70) | 65 (53, 75) | 59 (51, 66) | 1.02 (1.00, 1.04) | 0.027 | 0.99 (0.94, 1.04) | 0.636 |
| Male, n(%) | 118 (59.6) | 39 (59.1) | 79 (59.8) | 0.97 (0.51, 1.86) | >0.999 | — |  |
| Underlying medical condition(s) ^a^, n(%) |  |  |  |  |  |  |  |
| Steroid exposure, any | 49 (24.7) | 26 (39.4) | 23 (17.4) | 3.06 (1.49, 6.34) | 0.001 | 4.28 (0.40, 70.49) | 0.261 |
| Steroid exposure, prolonged^b^ | 24 (12.1) | 16 (24.2) | 8 (6.1) | 4.91 (1.85, 14.16) | <0.001 | 0.27 (0.02, 2.27) | 0.245 |
| Diabetes mellitus | 55 (27.8) | 17 (25.8) | 38 (28.8) | 0.86 (0.41, 1.75) | 0.737 | — |  |
| Use of immunosuppressants other than steroid^c^ | 34 (17.2) | 19 (28.8) | 15 (11.4) | 3.13 (1.38, 7.24) | 0.004 | 1.37 (0.21, 9.34) | 0.738 |
| Autoimmune disease | 34 (17.2) | 17 (25.8) | 17 (12.9) | 2.34 (1.03, 5.32) | 0.029 | 0.25 (0.02, 2.21) | 0.224 |
| Chronic liver disease^d^ | 44 (22.2) | 18 (27.3) | 26 (19.7) | 1.53 (0.72, 3.21) | 0.277 | — |  |
| Chronic kidney disease^e^ | 29 (14.6) | 14 (21.2) | 15 (11.4) | 2.10 (0.87, 5.03) | 0.087 | — |  |
| Solid organ malignancy | 60 (30.3) | 11 (16.7) | 49 (37.1) | 0.34 (0.15, 0.74) | 0.003 | 0.28 (0.03, 2.08) | 0.220 |
| Hematologic malignancy | 17 (8.6) | 8 (12.1) | 9 (6.8) | 1.88 (0.60, 5.80) | 0.281 | — |  |
| Absence of immunocompromising conditions^f^ | 44 (22.2) | 12 (18.2) | 32 (24.2) | 0.70 (0.30, 1.53) | 0.369 | — |  |
| Symptoms and signs, n(%) |  |  |  |  |  |  |  |
| Fever | 69 (34.8) | 45 (68.2) | 24 (18.2) | 9.50 (4.63, 20.20) | <0.001 | 0.53 (0.07, 2.97) | 0.495 |
| Any neurologic manifestations^g^ | 80 (40.4) | 63 (95.5) | 17 (12.9) | 136.0 (38.2, 740.7) | <0.001 | 125.3 (16.1, 2346.3) | <0.001 |
| Laboratory investigations |  |  |  |  |  |  |  |
| WBC, k/μL, median (IQR) | 6.69 (5.07, 8.78) | 7.76 (5.22, 10.42) | 6.22 (5.07, 7.85) | 1.05 (1.00, 1.12) | 0.016 | 1.03 (0.87, 1.20) | 0.732 |
| Neutropenia, n(%) (N=196) | 7 (3.6) | 2 (3.0) | 5 (3.8) | 0.78 (0.07, 4.94) | >0.999 | — |  |
| Lympocytopenia, n(%) (N=196) | 55 (28.1) | 37 (56.1) | 18 (13.8) | 7.83 (3.75, 16.94) | <0.001 | 1.01 (0.19, 5.02) | 0.992 |
| Hyponatremia, n(%) (N=190) | 58 (30.5) | 38 (57.6) | 20 (16.1) | 6.97 (3.38, 14.83) | <0.001 | 1.60 (0.40, 6.71) | 0.504 |
| Cryptococcemia, n(%) (N=117) | 34 (29.1) | 25 (37.9) | 9 (17.6) | 2.82 (1.11, 7.74) | 0.023 | 0.31 (0.04, 1.96) | 0.229 |
| Positive culture(s) from extrapulmonary,  extracranial sites, n(%) | 24 (12.2) | 9 (13.6) | 15 (11.4) | 1.23 (0.45, 3.22) | 0.650 | — |  |
| SCRAG titer, median (IQR) (N=161) | 16 (0, ≥1024) | ≥1024 (64, ≥1024) | 0 (0, 16) | 1.56 (1.39, 1.78)^j^ | <0.001 | 1.72 (1.36, 2.30)^j^ | <0.001 |

^a^ Medical conditions presented by 5 or more patients were provided.

^b^ Prolonged steroid exposure was defined by a minimum dose of 0.3 mg/kg/day of prednisolone for more than 3 weeks according to EORTC/MSG consensus, 2008.

^c^ Recorded immunosuppressants other than steroid included azathioprine, bleomycin, chlorambucil, cisplatin, cyclophosphamide, doxorubicin, fluorouracil, ifosphamide, oxaliplatin, mercaptopurine, methotrexate, mycophenolic acid (MMF), vincristine.

^d^ Chronic liver disease was defined when there was evidence of chronic viral hepatitis or the presence of cirrhosis.

^e^ Chronic kidney disease was defined when there was evidence of kidney damage or estimated glomerular filtration rate (eGFR) below 60 ml/min/1.73m^2^ for at least 3 months according to KDIGO guideline, 2012.

^f^ Immunocompromising conditions indicated here included diabetes, chronic kidney diseases, cirrhosis of liver, autoimmune diseases, malignant diseases, use of steroid or other immunosuppressants, and hypogammaglobulinemia.

^g^ Neurologic manifestations included headaches, altered mental status, seizures, meningeal signs and focal neurologic signs.

^h^ Comparison group was composed of patients who had confirmed negative CSF study and who had a clinical course compatible with non-CNS cryptococcosis.

^i^ Variables with *p*-value <0.2 in univariate analysis were included in the multivariate analysis.

^j^ Odds ratio per 2-fold increment of sCRAG.

Abbreviations: CI, confidence interval; CNS, central nervous system; sCARG, serum cryptococcal antigen titer; WBC, white blood cell count.
