# Supplementary material for: Lumbar puncture for non-HIV-infected non-transplant patients with cryptococcosis: Should it be mandatory for all?
Source: PLoS One. 2019 Aug 22;14(8):e0221657. doi: 10.1371/journal.pone.0221657 (PMC6705819; doi:10.1371/journal.pone.0221657)
Supplement: S3 Table — (DOCX) [file pone.0221657.s003.docx]

S3 Table. Association of Various Neurologic Manifestations and CNS Involvement among Non-HIV-infected, Non-transplant Patients with Cryptococcosis by Univariable and Multivariable Analyses

|  | All  (N=93) | With CNS involvement  (N=66) | Without CNS involvement  (N=27) | Univariate analysis | | Multivariate analysis | |
| --- | --- | --- | --- | --- | --- | --- | --- |
|  |  |  |  | Odds ratio, 95% CI | *p*-value | Odds ratio, 95%CI | *p*-value |
| Symptoms, n (%) |  |  |  |  |  |  |  |
| Headache | 38 (40.9) | 36 (54.5) | 2 (7.4) | 14.62 (3.21, 137.73) | <0.001 | 12.30 (3.09, 83.55) | 0.002 |
| Altered mental status | 48 (51.6) | 37 (56.1) | 11 (40.7) | 1.84 (0.68, 5.14) | 0.253 | — |  |
| Seizures | 6 (6.5) | 4 (6.1) | 2 (7.4) | 0.81 (0.11, 9.47) | >0.999 | — |  |
| Signs, n (%) |  |  |  |  |  |  |  |
| Meningeal signs | 15 (16.1) | 14 (21.2) | 1 (3.7) | 6.90 (0.95, 306.75) | 0.059 | 3.06 (0.40, 62.98) | 0.337 |
| Focal neurologic signs | 30 (32.3) | 26 (39.4) | 4 (14.8) | 3.69 (1.08, 16.36) | 0.028 | 4.02 (1.23, 16.01) | 0.030 |

Abbreviations: CI, confidence interval; CNS, central nervous system.
